# Supraclavicular approach for neonatal brachial plexus palsy

**DOI:** 10.3171/2022.10.FOCVID22109

**Published:** 2023-01-01

**Authors:** Yamaan S. Saadeh, Whitney E. Muhlestein, Lynda J. S. Yang, Brandon W. Smith

**Affiliations:** 1 Department of Neurosurgery, University of Michigan, Ann Arbor, Michigan; and; 2 Department of Neurologic Surgery, Duke University, Durham, North Carolina

**Keywords:** neonatal brachial plexus palsy, nerve reconstruction, supraclavicular approach

## Abstract

Neonatal brachial plexus palsy describes injury to the brachial plexus in the perinatal period, resulting in motor and sensory deficits of the upper arm. Nerve reconstruction, including graft repair and nerve transfers, can be used to restore function in patients whose injury does not respond to conservative management. Despite the availability of these techniques, 30%–40% of children have lifelong disability, reflecting a 10-fold underutilization of surgery. Here, the authors demonstrate a supraclavicular approach for brachial plexus exploration, as well as a spinal accessory to suprascapular nerve transfer for restoration of shoulder abduction and external rotation.

The video can be found here: https://stream.cadmore.media/r10.3171/2022.10.FOCVID22109

## Transcript

Neonatal brachial plexus palsy has an incidence of approximately 1 out of 1000 live births. This is a similar incidence that we see in cerebral palsy. The most common cause of the palsy itself is traction to the shoulder, which causes tension on the nerves of the brachial plexus. The most common presentation is an upper trunk injury, also referred to as an Erb’s palsy. Prior to the 1980s, management of the brachial plexus palsy was generally conservative. Since the 1980s, there have been some considerable changes in the way that we manage brachial plexus palsy. The initial form of treatment of the brachial plexus palsy was nerve grafting. However, in the 1990s, the development of nerve transfers presented an alternative approach to the management of these injuries. Thankfully, a majority of these patients recover. However, 30%–40% of the patients will continue to have persistent deficits into adulthood.

### 1:11 Case Presentation.

In this specific case, a 7-month-old female presented with right upper-extremity weakness from birth. The pregnancy was complicated by gestational diabetes. The mother recalls a difficult delivery and states that ever since the baby was born, she could only move her hand. She underwent an intensive, 3-month course of occupational therapy without any significant improvement.

### 1:31 Electrodiagnostics.

Electrodiagnostic testing revealed an upper and middle trunk palsy with sparing of the lower trunk.

### 1:31 Imaging.

Preoperative imaging demonstrated edema of the brachial plexus with disruption of the C6 nerve root. The ultrasound demonstrated normal diaphragmatic motion with an abnormal shoulder and glenohumeral dysplasia as well as shoulder subluxation.

### 1:49 Operative Planning.

The operative considerations included nerve transfers versus nerve grafting, the presence or absence of shoulder abnormalities such as subluxation, and the use of neuromonitoring intraoperatively.

### 1:59 Patient Positioning.

To prepare for the supraclavicular exposure and brachial plexus surgery, we prefer a supine position with the head flexed and away. As you can see in this picture, an Ambu bag is often used for the support of the head during surgery. We place a small piece of padding in between the chin and the shoulder to prevent kinking of the vasculature. In cases where we are utilizing nerve transfers, we like to keep the operative arm completely draped in order to utilize multiple positions for the other nerve transfers. We use gauze and padding to support the operative arm during surgery.

### 2:29 Patient Marking and Operative Landmarks.

Prior to the incision, we like to mark all of our surgical landmarks. We then plan an incision approximately 1 cm above the clavicle going from the lateral edge of the sternocleidomastoid to the insertion of the trapezius on the clavicle.

### 2:49 Surgical Incision.

An incision is made in the skin down to the layer of the superficial fat. We like to use a mixture of retraction and blunt dissection to expose the platysma.

### 2:57 Splitting the Platysma.

Once the platysma is exposed, we like to split the fibers until we reach the posterior aspect of the platysmal fascia. You can see a layer of supraclavicular fat. We utilize this plane to perform a subplatysmal dissection from end to end of the incision. The supraclavicular nerves as well as the external jugular vein live in this plane.

### 3:17 Exposure of the Spinal Accessory Nerve.

To expose the spinal accessory nerve, move to the lateral aspect of your incision. You should be able to see the insertion of the trapezius on the clavicle. If you utilize the deep fascia of the clavicular head of the trapezius, you should be able to separate the fat from this plane, which should help you find the location of the spinal accessory nerve. You can also use intraoperative stimulation, which is demonstrated here. If you happen to find yourself deep within muscle, you have likely gone too deep. The nerve itself often has somewhat of a serpiginous appearance.

### 3:46 Isolation and Mobilization of the Spinal Accessory Nerve.

At this point, you should work to isolate and free the nerve from the surrounding tissues. It is important here to spend considerable amount of effort in exposing the spinal accessory nerve, as it will be your donor and any length will be appreciated during the second part of the procedure. Once you have isolated the spinal accessory nerve, you can then move to exposing the brachial plexus itself.

### 4:07 Disconnecting the Sternocleidomastoid From the Clavicle.

At the medial aspect of your incision, you can follow the lateral edge of the sternocleidomastoid down to the clavicle. It is at this point that we like to utilize bipolar cautery to remove a portion of the lateral aspect of the sternocleidomastoid from the clavicle. This will help in visualization.

### 4:23 Identification and Division of the Omohyoid.

At this point, you can start to mobilize the fat pad from the medial aspect of your incision. You will often encounter the omohyoid at this level. Isolation of this muscle can aid in retracting both the fat pad as well as the medial aspect of the sternocleidomastoid that you just divided.

### 4:39 Mobilization of the Supraclavicular Fat Pad.

At this point, you can often feel a neuromatous formation of the injury below. In some injuries, it is difficult to feel the separation between the anterior scalene and the brachial plexus. When moving toward mobilizing the fat pad, it is important that you go straight down toward the scalene and brachial plexus. If you end up drifting medially, you can find yourself encountering the carotid sheath.

### 5:01 Identification of the Neuromatous Brachial Plexus and Anterior Scalene.

After exposure of the supraclavicular fat pad, you can now see the underlying neuroma as well as the anterior scalene. You can follow the lateral edge of the upper trunk to find the suprascapular nerve. You should then move toward exposing the brachial plexus proximally, as well as the phrenic nerve.

### 5:17 Exposure of the Phrenic Nerve. The phrenic nerve itself lives on top of the anterior scalene.

As it moves from cranial to caudal, it goes lateral to medial, which is unique in its identification. You can use the phrenic nerve to help find the location of the upper trunk or the C5 nerve root.

### 5:31 Exposure of the Suprascapular Nerve.

Once you have located the upper trunk, follow the lateral aspect of the upper trunk until you see the first branch coming off laterally. This should be the suprascapular nerve. When isolating the suprascapular nerve for nerve transfer, you often have to mobilize it more proximally. As this is a neonatal brachial plexus surgery, you can sometimes see stimulation of the suprascapular nerve. This phenomenon has been described and is often called "luxury innervation." It is unlikely that this residual innervation will cause any evidence of recovery.

### 5:59 Coaptation.

We like to utilize background material to facilitate the coaptation between the spinal accessory nerve and the suprascapular nerve. In our practice, we like to utilize 10-0 epineurial stitches in order to complete the coaptation. We also prefer to back this coaptation up with a layer of tissue glue. Some practices utilize a glue-only coaptation and also use conduits. This is up to personal preference and practice patterns at your local institution.

### 6:25 Closure.

During closure we like to reapproximate the omohyoid, as we have already left tendon stitches in it during the previous dissection. We often like to mobilize the fat pad and place it back over the brachial plexus to facilitate its protection. We then close in layers, including the platysma and subcutaneous tissues, and close the top layer with skin glue.

### 6:43 Additional Nerve Transfers.

The treatment of an upper trunk palsy commonly requires a reconstruction of shoulder external rotation, shoulder abduction, and elbow flexion. Spinal accessory to suprascapular nerve transfer aims to reconstruct external rotation and initiation of abduction of the shoulder. This particular patient also underwent a radial to axillary nerve transfer to reinnervate the deltoid, as well as an ulnar to musculocutaneous, otherwise known as an Oberlin nerve transfer, to reinnervate the bicep.

### 7:10 Postoperative Botox Injections and Bracing.

For patients with contractures of the pectoralis or subluxation of the shoulder, we use Botox injections into the pectoralis muscle as well as replacement of the humeral head into the glenohumeral joint and placement of a brace prior to full emergence from anesthesia.

### 7:25 Follow-Up.

The patients are brought back to clinic every week for 6 weeks, as they do wear a Spica brace continuously during this time. These visits are helpful to maintain the cleanliness of the brace as well as to monitor the incisions. After the brace is removed at the 6-week point, the children can then start their extensive rehabilitative program.

### 7:41 References^1–10^

We hope that this video has been helpful for you in seeing how we perform a supraclavicular exposure for a neonatal brachial plexus repair, in this case a spinal accessory to suprascapular nerve transfer, which was the first portion of an upper trunk brachial plexus repair.

## Acknowledgments

We thank Miriana Popadich.

## Disclosures

The authors report no conflict of interest concerning the materials or methods used in this study or the findings specified in this publication.

## Author Contributions

Assistant surgeon: Muhlestein. Editing and drafting the video and abstract: Smith, Saadeh, Muhlestein. Critically revising the work: Smith, Saadeh, Muhlestein. Reviewed submitted version of the work: Smith, Saadeh, Muhlestein. Approved the final version of the work on behalf of all authors: Smith. Supervision: Smith, Yang.
